# Honeycomb Column Thin-Walled Structure Design and Mechanical Properties of Ti Alloy Fabricated through Selective Laser Melting

**DOI:** 10.3390/ma16196552

**Published:** 2023-10-04

**Authors:** Shenghua Zhang, Jingshuai Shi, Bin Liu, Zhonghua Li

**Affiliations:** 1 School of Materials Science and Engineering, North University of China, Taiyuan 030051, China; shenghuazhang@nuc.edu.cn (S.Z.); liubin3y@nuc.edu.cn (B.L.); 2 School of Mechanical Engineering, North University of China, Taiyuan 030051, China; 18406583495@163.com

**Keywords:** selective melting, titanium alloy, numerical simulation, thin-walled structure

## Abstract

A honeycomb column thin-walled structure (HCTS) was designed and the relative density was calculated for numerical simulation. The HCTS samples were fabricated via selective laser melting (SLM). The numerical simulation and a three-point bending test were conducted to evaluate the mechanical properties of the HCTS made of Ti6Al4V. The findings of the numerical simulation demonstrated that the HCTS had a stronger resistance to deformation and a maximum loading force 30% higher than the equivalent solid thin-walled structure (ESTS). The mechanical performance of the HCTS as determined by the three-point bending test was mostly comparable with the numerical simulation. The maximum loading force of the experimental HCTS050-E thin-walled structure was 1200 N higher than that of HCTS050-S. The numerical simulation can provide theoretical guidance for the SLM processing of HCTSs.

## 1. Introduction

Many academics have studied thin-walled constructions since the beginning of the 1960s, particularly thin-walled pipe parts. Parts often fail in stress concentrations, and when under high loads, these localized locations are more vulnerable to failure. The local stress is significantly more than the nominal stress. The stress concentration typically takes place where the cross-sectional geometry suddenly changes. This significant mechanical element contributes to structural collapse. Therefore, the cross-section of a circle is the most ideal form in nature since there is no stress accumulation in it [1,2,3,4]. The concept of additive manufacturing of bionic honeycomb column thin-walled structures proposed by Hao [5] was realized through a combination of experiments and numerical simulations [6]. The structure and mechanical properties of Ni-15Fe-5Mo thin walls with different wall thicknesses and overhanging angles were studied [7]. It was found that many micrometer- or sub-micrometer-sized cellular structures were generated within the grains, which typically grew through more than one melt pool, and that the thin-walled samples with lower wall thickness processed finer grain sizes. A series of novel three-dimensional curved-walled mixed-phase honeycombs were proposed by Yang et al. [8]. It was reported that the proposed honeycombs displayed efficient progressive folding modes and desirable mechanical properties, and the gap between peak and mean stresses effectively narrowed. The thickness gradient design made the stiffness distribution more efficient and further improved the out-of-plane crashworthiness. The above studies indicate that honeycomb column thin-walled structures have superior tensile and compressive properties, energy absorption characteristics, and formability compared to square or multi-tube structures.

Ti6Al4V alloy is a low-density, high-strength alloy material with good corrosion resistance, heat resistance, plasticity, toughness, formability, weldability, and biocompatibility, making it an ideal structural material for aerospace components [9]. However, due to its high melting point, low thermal conductivity, low modulus of elasticity, and susceptibility to cracking, there are problems in the traditional machining process, such as low dimensional accuracy of the parts, difficulty in machining and molding, severe tool wear, and energy consumption, resulting in the low utilization of titanium alloy materials [10]. The utilization of SLM technology to print titanium alloy components with high dimensional accuracy eliminates tool wear and improves the material utilization; therefore, SLM technology has been widely used in the fabrication of titanium alloy components. As a lightweight and high-performance structure, titanium alloy thin-walled parts are widely used in the aerospace industry. However, the thin-walled titanium structure is susceptible to bending deformation during SLM processing, making it difficult to ensure forming quality.

Many researchers have studied the mechanical properties of titanium alloy thin-walled components. A 3D transient fully coupled thermomechanical model was constructed to investigate the distortion and residual stress in Ti-6Al-4V plates produced using electron beam additive manufacturing [11]. The plate distortion was measured for comparison with the model simulations. The results showed that the simulated distortion and residual strains were in good agreement with the experimental results. The surface morphology, microstructure, and tensile properties of the electron-beam-melted titanium parts were evaluated [12]. The findings indicated that microstructure was influenced by wall thickness, and for thin-walled structures, variations in surface roughness might dominate tensile properties. A force model of machining titanium alloy thin-walled parts was developed based on the actual geometry and abrasive characteristics of the CBN grinding head [13]. It was reported that the surface roughness of the vertical side wall surface and the surface micro-hardness are determined by several key parameters including spindle speed, feed rate, and cutting depth in the grinding process based on analysis of experiments. Finite element modeling of the ballistic impact of inserts containing titanium structures was reported and the models provide a proper representation of the actual behavior of materials [14]. Most of the research has focused on the optimization of SLM parameters of the thin-walled structures, while there are fewer studies on the design of titanium thin-walled structures and the evaluation of deformation resistance.

In this work, an HCTS was designed and the relative density was calculated for numerical simulation. The loading analysis was completed to establish the structure’s finite element modeling for the three-point bending test. The three-point bending test processes for the honeycomb thin-walled models were simulated and analyzed. The improvement in mechanical properties of the HCTS compared to the ESTS was demonstrated through the numerically simulated three-point bending test. The honeycomb column thin-walled structural samples made of Ti6Al4V were fabricated via SLM and the mechanical properties were evaluated to validate the numerical simulation results. The novelty of this study lies in the evaluation of the bending deformation resistance of the HCTS through the three-point bending test results of simulated HCTS and ESTS models, and experimental HCTS samples.

## 2. Honeycomb Column Thin-Walled Structure Model

Honeycomb structures are composed of multiple cell elements assembled in a two-dimensional plane and are generally subjected to axial pressure. Various modes of honeycomb structures exhibit similar forms of compression collapse under axial pressure, with some degree of boundary effects [15,16,17,18,19,20].

**Figure 1:**
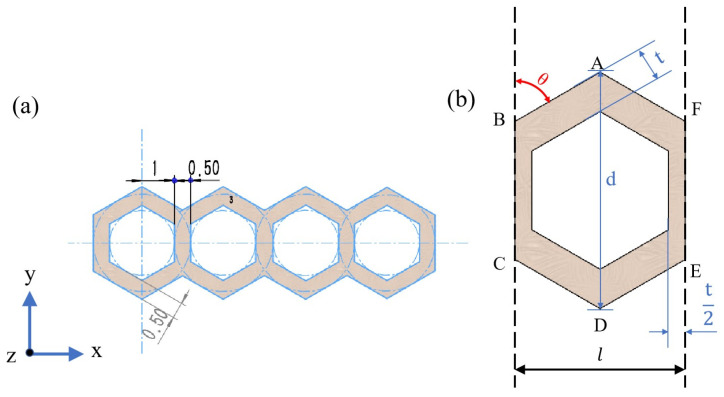
(**a**) Schematic diagram of Y-cell honeycomb structure. (**b**) A representative cell of honeycomb structure. The blue arrows are coordinate axes.

The most basic cellular structure of the honeycomb is the V-cell, which is categorized into acute, right, and obtuse angle cells based on the angle of the V-cell. Wierzbicki [21] discovered and established the nonductile angle cell, which initiated the study of the classical model of polygonal thin-walled structures. The same node can consist of an arbitrary number of cell elements at the same node, determined by the angle of the basic V-cell. Each cellular element of the honeycomb is a regular hexagon, and the angle of the V-cell is 120°, which presents a Y-shape and is called a Y-cell honeycomb structure, as shown in Figure 1a.

A representative cell taken from Figure 1a is shown in Figure 1b. The thickness of the thin-walled structure, *d*, can be expressed by Equation (1).

(1) $$d=\frac{2\left(l+2t\right)cos\theta +l+t}{2sin\theta }$$

where *t* is the wall thickness of the cellular structure’s cell element, which is the spacing between the inner wall and the outer wall. A single-cell element has a wall thickness of *t*/2 in the x-direction. *θ* is the angle between the cell element’s ledge AB and the ledge BC, and it ranges from 45° to 60°. *l* denotes the distance between the ledge BC and the ledge EF.

By calculating the relative density of the honeycomb structure, the mechanical properties such as structural stiffness, strength, and toughness can be analyzed. The area of the walls of a single cell element is denoted as *S_xy_*, which can be calculated using Equation (2). *S_α_* is the total area enclosed by A–E points, which can be calculated using Equation (3). Equations (1)–(3) were derived from the geometry of the model. The relative density of the cellular structure was calculated using Equation (4) [22].

(2) $$S_{xy}=\frac{t^{2}cos\theta +9tl}{2sin\theta }$$

(3) $$S_{\alpha }=\frac{2\left(l^{2}+2tl\right)cos\theta +l^{2}+tl}{2sin\theta }$$

(4) $$\rho =\frac{S_{xy}}{S_{\alpha }}=\frac{t^{2}cos\theta +9tl}{2\left(l^{2}+2tl\right)cos\theta +l^{2}+tl}$$

## 3. Loading Analysis of Y Honeycomb Column Structure

**Figure 2:**
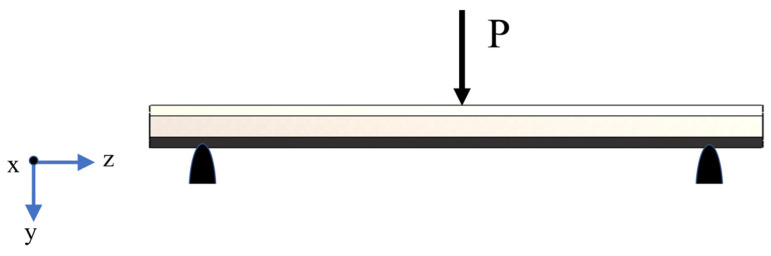
Stress condition of honeycomb structure. The blue arrows are coordinate axes.

Since the Y-cell honeycomb structure is a single-layer honeycomb structure, as shown in Figure 1a, when the bending load P is applied to its middle part, its force is as shown in Figure 2. Supports were added on both sides of the bottom of the thin-walled structure, and displacement constraints vertically downward along the *z*-axis were applied to the middle part. The three-point bending test was used to evaluate the mechanical properties of the thin-walled honeycomb structure.

**Figure 3:**
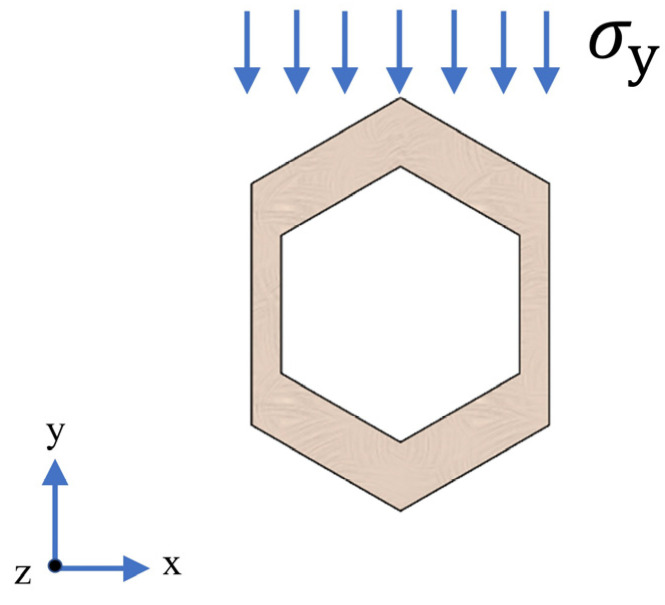
Stress condition of a cross-section of a single cell.

Since the direction of the applied load is parallel to the cross-section and there is no remaining external force, vertical wall plate deformation is considered. According to the periodicity of the structure, a single cell is taken from Figure 1a, and the force on the single cell element is analyzed by restraining its rotation along the *y*-axis and *z*-axis and its translation along the *x*-axis and *z*-axis, and retaining the rotation along the *x*-axis and translation along the *y*-axis. The effective cross-sectional area of the cell element in the y-direction, *S_y_*, and the positive bending stresses *σ_y_* are expressed as Equation (5) and Equation (6), respectively [23].

(5) $$S_{y}=h\times l$$

(6) $$\sigma_{y}\ll \sigma_{MAX}=\frac{My_{MAX}}{I_{x}}=\frac{M}{W_{x}}$$

where *h* is the height of the honeycomb structure in the along-axis direction and *l* is the total length of a single cell, as shown in Figure 1b; *M* denotes the bending moment in the span; *I_x_* is the moment of inertia of the cell cross-section concerning the *x*-axis; and *W_x_* is the coefficient of the bending cross-section. The forces on the cell at the intermediate cross-section of the HCTS are shown in Figure 3.

## 4. Numerical Simulations

**Figure 4:**
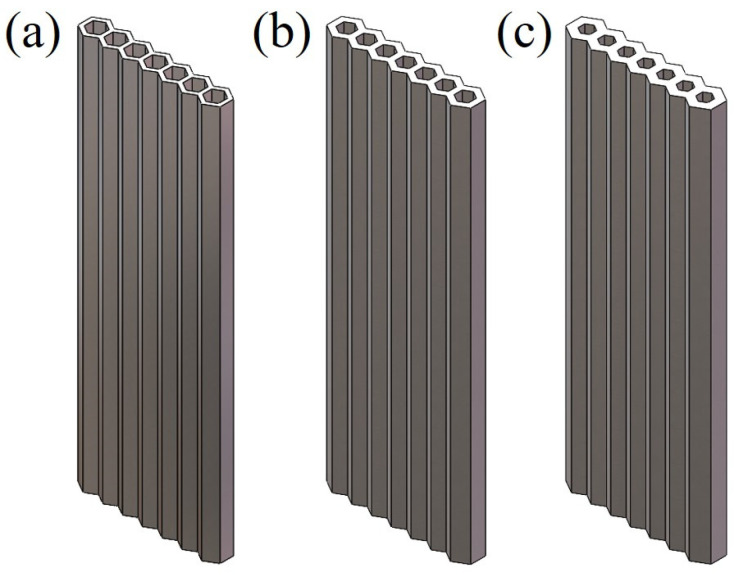
Honeycomb column thin-walled structure models. (**a**) HCTS050, (**b**) HCTS075, (**c**) HCTS100.

In order to verify the theoretical model designed using the above method, the honeycomb column thin-walled parametric model was established using Solidworks and numerically analyzed by using Ansys Workbench. The geometric model is shown in Figure 4, and the global coordinate system adopted a right-handed coordinate system, with the *x*-axis horizontally to the right, the *y*-axis vertically upward, and the *z*-axis perpendicularly outward from the plane. A total of three honeycomb column thin-walled structures with different wall thicknesses were modeled, and the geometric parameters were calculated using Equations (1)–(6) above, as displayed in Table 1. The honeycomb column thin-walled structures with different wall thicknesses were named HCTS050, HCTS075, and HCTS100, respectively. *T* is the thickness of a single modeled cell and *S_xy_* is the actual cross-sectional area of the model. *S_α_* is the effective cross-sectional area of the model and *ρ* is the relative density. Finite element meshing was performed for the three models. Titanium alloy Ti6Al4V was selected as the material for the simulation. The physical parameters of Ti6Al4V are listed in Table 2. Because the effect of the mesh density on the calculation results in the calculation of the extended finite element method is less, the mesh size is uniformly adopted as the approximate global size of 0.0005 m [24].

**Figure 5:**
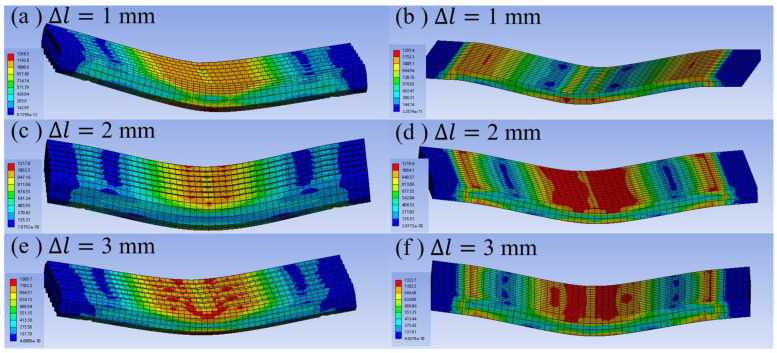
Stress nephograms of the HCTS075 model (**a**,**c**,**e**) and the corresponding ESTS model (**b**,**d**,**f**) with different displacements of pressure head.

The three-point bending test processes for the honeycomb column thin-walled models and the equivalent ESTS models were simulated. The stiffness behavior of HCTS and ESTS models was set to be flexible and that of the pressure head was set to be rigid. Since the numerical simulation results for different wall thicknesses were similar, the stress nephograms for the HCTS075 thin-walled model and the corresponding solid model are shown on the left side and right side of Figure 5, respectively, with different displacements of the pressure head. The tensile test was conducted to obtain the ultimate tensile strength and the results showed that it was around 1200 MPa. Thus, bending failure was considered when a stress of more than 1200 MPa appeared at the bottom of the models. As shown in Figure 5a,c,e, a large stress area with red color appeared the earliest at the junctions of corners and the pressure head in honeycomb thin-wall models. The stress was uniformly distributed on every cell. When the pressure head displacement reached 1 mm, the maximum stress of 1287 MPa appeared in the corners, and the corner contacting with the pressure head appeared to be cracked. This was because the contact mode of corners with the pressure head was line contact, which resulted in excessive stress. When the displacement was 2 mm, the maximum stress was 1218 MPa and scattered large stress areas appeared in the superficial cells of the model. A large stress area also appeared at the bottom of the model, which was due to the bottom being subjected to excessive tensile displacement. When the displacement further increased to 3 mm, the maximum stress reached 1310 MPa, which appeared at the top corner of the cells. The maximum stress in the body of the cells was 1102 MPa. The large stress area spread to the side of the cells, and the stress distribution of the overall model showed that the tangent to the pressure head and the support displayed the large stress area, which radiated to the surrounding area to narrow down. The model showed a V-shape deformation but no bending failure.

As shown in Figure 5b, the stress nephograms of the equivalent solid thin-walled model indicated that the maximum stress was 1297 MPa and it appeared at the supports and the middle of the model. With the displacement increased, a large stress appeared at the areas that were tangential to the pressure head and spread to the sides of the model, showing a butterfly-like radiation decay. The maximum stress was 1220 MPa. There were also large stress areas at the supports. When the displacement reached 3 mm, the stress at the bottom of the model’s middle part was higher than 1200 MPa, and severe deformation and bending failure occurred.

**Figure 6:**
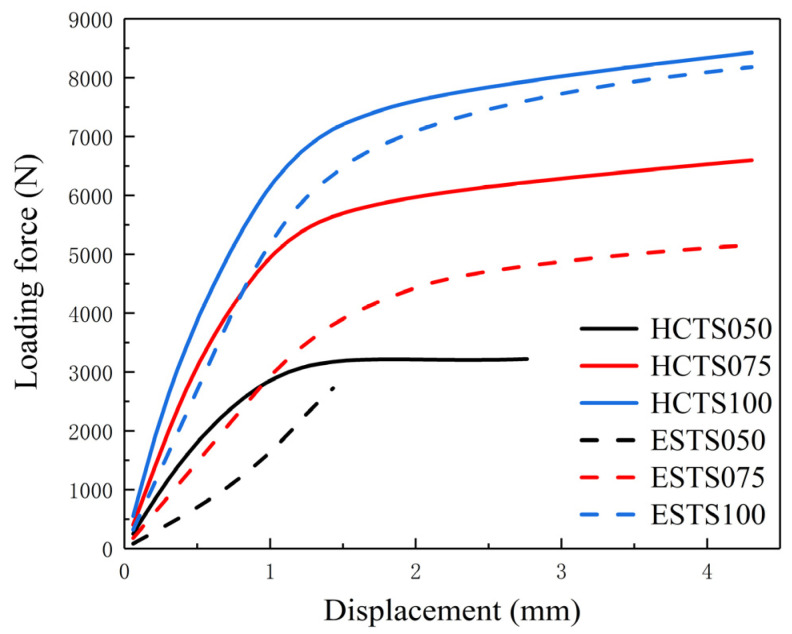
Curves of loading force versus the displacement of the pressure head during the numerical simulation of three-point bending for HCTS and ESTS series.

The maximum stress values of the HCTS series and ESTS series during deformation were close, but the resistance to deformation of the HCTS series was much higher. The curves of loading force versus the displacement of the pressure head during the numerical simulation of three-point bending are plotted in Figure 6. Solid lines indicate HCTS models and dashed lines indicate the equivalent ESTS models. It can be seen that under the same displacement, the loading force of the HCTS series was larger than that of the ESTS series. The most significant improvement between the HCTS and ESTS was shown by the results of HCTS075. The maximum loading force of HCTS075 was 30% higher than that of ESTS075. As the wall thickness increased, the difference between their loading force decreased, which meant the larger the wall thickness was, the similar mechanical performance they exhibited. This may be due to the fact that when the wall thickness was thicker, the holes became smaller and they approached those of the solid models. The structural benefits diminished. It was noted that the equivalent ES050 fractured when the displacement reached 1.3 mm, which indicated that the wall thickness was a major factor limiting the mechanical properties of the solid thin-walled structure. However, when the HCTS050 was subjected to the bending deformation, the displacement reached 2.7 mm. Therefore, the structural advantages of the HCTS were more obvious when the wall thickness was smaller.

## 5. Fabrication and Mechanical Properties

**Figure 7:**
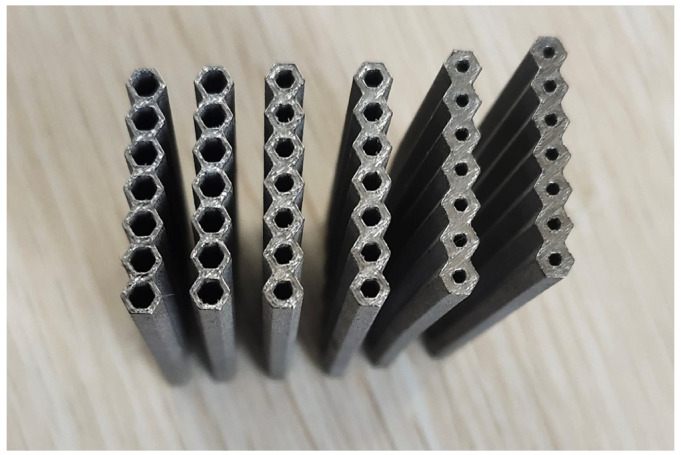
Honeycomb column thin-walled structure samples printed with Ti6Al4V alloy powder via SLM.

The raw material was a spherical powder of the Ti6Al4V alloy that had been prepared via the vacuum atomization method. The powder’s particle size was smaller than 64.35 µm, and the median particle size was 40.56 µm, which met the powder spreading requirements for SLM manufacturing. EP-M150 SLM equipment with a fiber laser (beam spot diameter, 70 µm; maximum scanning speed, 8 m/s) was used to print the above models. The samples of 50 mm in length were built and the parameters for the cross-section are illustrated in Figure 1. Two samples were printed for each HCTS series and the photos of as-printed samples are shown in Figure 7. The SLM processing parameters of the samples included a laser power of 180 W, a scanning speed of 1000 mm/s, a hatch spacing of 0.12 mm, and a powder thickness of 30 µm. It was noted that the inner wall roughness of HCT050 was smoothest. This was because the area of the inner wall increased with the decrease in wall thickness, and the cooling rate of HCT050 was higher than that of HCT100. The slower cooling rate of HCT100 led to a serious adhesion of powders.

Three-point bending tests were carried out on the above as-printed samples using a universal testing machine with a support distance of 40 mm, a preload value of 100 N, and a loading rate of 0.2 mm/min. The loading was stopped until the specimen was damaged or the loading was reduced from the peak value to 50% of the maximum loading.

**Figure 8:**
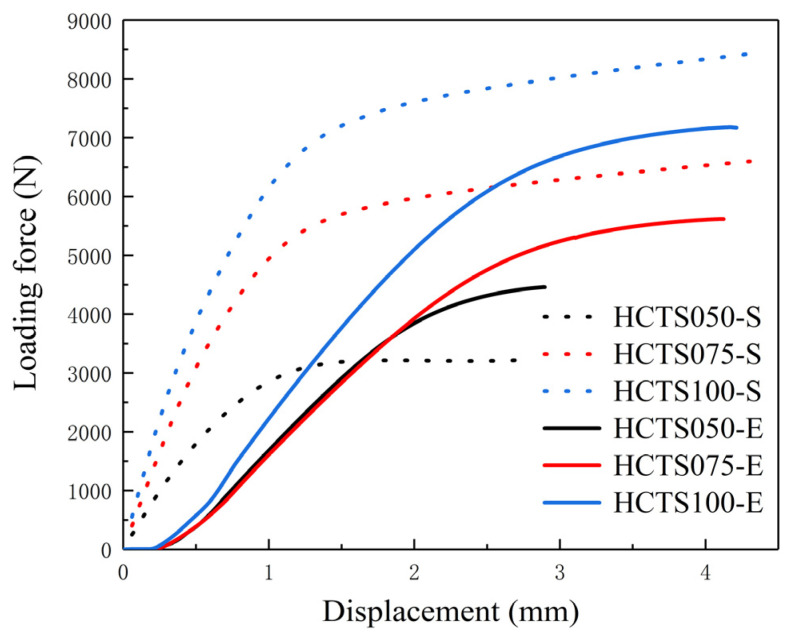
The comparison of numerical and experimental results of loading force versus the displacement of the pressure head during the three-point bending test for the HCTS series.

The comparison of simulated (HCTSXXX-S) and experimental (HCTSXXX-E) results of the loading force versus the displacement of the pressure head during the three-point bending tests is shown in Figure 8. It can be seen that curves of HCTS050-E and HCTS075-E almost coincided before the displacement reached 2 mm. When the displacement continued increasing, there was a large increase in the force loading of the HCTS075-E sample, while the HCTS050-E sample cracked after a further increase of 1000 N. The experimental loading force–displacement curves were in accordance with those of the numerically simulated ones. The numerically simulated HCTS100-S had a loading force of 8300 N when the pressure head displacement reached 4 mm, while HCTS100-E cracked with 7300 N in the three-point bending test. The maximum loading force of HCTS075-S was 1000 N higher than that of HCTS075-E, which had the same trend as the HCTS100 thin-walled structure. However, the maximum loading force of the experimental HCTS050-E thin-walled structure was 1200 N higher than that of HCTS050-S, which was opposite to the results of HCTS075 and HCTS100. This may be due to the finer grains of the HCTS050 thin-walled structure [7] and the roughness of the inner wall [12,13].

## 6. Conclusions

A honeycomb column thin-walled structure was designed and the relative density was calculated for numerical simulation. The HCTS samples were fabricated via selective laser melting. Numerical simulations and experiments of three-point bending tests were conducted to evaluate the mechanical properties of the Ti6Al4V-made HCTS. The conclusions are as follows.

(1) The simulated stress nephograms showed that the bending deformation resistance of the HCTS was higher than that of the ESTS. The ESTS can be replaced with the HCTS to improve its bending resistance.
(2) The results of the numerical simulation of three-point bending showed that the maximum loading force of the HCTS was higher compared with the ESTS. The most significant improvement between the HCTS and ESTS was shown by the results of HCTS075. The maximum loading force of HCTS075 was 30% higher than that of ESTS075.
(3) The numerical simulation results of the HCTS were basically consistent with the mechanical performance obtained from the three-point bending test. The numerical simulation can provide theoretical guidance for the SLM processing of the HCTS.
(4) For a more comprehensive evaluation of the HCTS, the mechanical behavior under compression might be investigated in the future.

## Figures and Tables

**Table 1:** Parameters of the honeycomb column thin-walled structure models.

Models	*t* (mm)	*θ* (°)	*S_xy_* (mm^2^)	*S_α_* (mm^2^)	*ρ* (%)
HCTS050	0.50	60	24.47	54.66	44.77
HCTS075	0.75	60	35.69	68.43	52.16
HCTS100	1.00	60	46.77	74.74	62.58

**Table 2:** Physical parameters of Ti6Al4V.

Materials	Elasticity Modulus (GPa)	Poisson Ratio	Density (kg/m^3^)
Ti6Al4V	110	0.34	4400

## Data Availability

Not applicable.
